# Simu-D: A Simulator-Descriptor Suite for Polymer-Based Systems under Extreme Conditions

**DOI:** 10.3390/ijms222212464

**Published:** 2021-11-18

**Authors:** Miguel Herranz, Daniel Martínez-Fernández, Pablo Miguel Ramos, Katerina Foteinopoulou, Nikos Ch. Karayiannis, Manuel Laso

**Affiliations:** Institute for Optoelectronic Systems and Microtechnology (ISOM) and Escuela Técnica Superior de Ingenieros Industriales (ETSII), Universidad Politécnica de Madrid (UPM), José Gutierrez Abascal 2, 28006 Madrid, Spain; miguel.herranzf@upm.es (M.H.); daniel.martinez.fernandez@upm.es (D.M.-F.); pm.ramos@alumnos.upm.es (P.M.R.); k.foteinopoulou@upm.es (K.F.)

**Keywords:** Monte Carlo, atomistic simulation, molecular simulation, hard sphere, extreme conditions, confinement, nanocomposites, cluster, crystallization, atomic structure, packing, semi-flexible polymers, order parameter

## Abstract

We present Simu-D, a software suite for the simulation and successive identification of local structures of atomistic systems, based on polymers, under extreme conditions, in the bulk, on surfaces, and at interfaces. The protocol is built around various types of Monte Carlo algorithms, which include localized, chain-connectivity-altering, identity-exchange, and cluster-based moves. The approach focuses on alleviating one of the main disadvantages of Monte Carlo algorithms, which is the general applicability under a wide range of conditions. Present applications include polymer-based nanocomposites with nanofillers in the form of cylinders and spheres of varied concentration and size, extremely confined and maximally packed assemblies in two and three dimensions, and terminally grafted macromolecules. The main simulator is accompanied by a descriptor that identifies the similarity of computer-generated configurations with respect to reference crystals in two or three dimensions. The Simu-D simulator-descriptor can be an especially useful tool in the modeling studies of the entropy- and energy-driven phase transition, adsorption, and self-organization of polymer-based systems under a variety of conditions.

## 1. Introduction

The development of new materials with enhanced properties is one of the most interesting and important topics in research in materials science and engineering. To achieve this ambitious goal, one has to relate the behavior of atoms and molecules to the macroscopic properties of the end material. In this perspective, molecular simulation is of paramount importance, since it allows the study of materials at the atomistic/molecular level without needing an experimental process, which, in specific cases, can become expensive, time consuming, and environmentally hazardous. Over the years, different molecular simulation techniques and methodologies have risen to answer relevant questions of general atomic and particulate systems [1,2,3,4,5].

A system composed of macromolecules is a very challenging case from the perspective of molecular simulation. This stems from the fact that polymers are characterized by a wide spectrum of characteristic time and length scales. Their simulation can become prohibitively difficult when very long and well-entangled chains are involved due to the very slow dynamics. Added to this is the fact that atomistic simulations have to take into full account the chemical constitution of the repeat units and the corresponding bonded and non-bonded interactions. To address this problem, a large amount of different molecular simulation methods has been developed and constantly improved over the last decades. The choice of simulation approach/scheme depends on the system/phenomenon, its physical-chemical details, size, and properties of interest. For example, Molecular Dynamics (MD) provides dynamical information at the local level of segments and global one of chains. However, as it follows the evolution of the equations of motion in time, it can be too slow to be effective when very long chains are involved. Monte Carlo (MC), by resorting to different stochastic algorithms (“moves”), can offer rapid equilibration at all length scales. However, MC cannot provide any information about the real dynamics. Accordingly, it is not uncommon for different techniques to be combined together into powerful hierarchical modeling approaches. The MD approach is widely used when dynamical or temporal evolutions are of interest. One of the most widely used software packages for the simulation of synthetic polymers is LAMMPS [6], which has been further used in other tools such as Polymatic for the polymerization of amorphous polymers [7]. Regarding the modeling of biomolecular systems, NAMD [8] and GROMACS [9] are two of the most popular simulation software, both placing special emphasis on parallelization in order to enhance performance. Other relevant open MD software suites are ms2 [10], to extract thermodynamical properties of homogeneous fluids using hybrid parallelization on MPI and OpenMP [11]; MOLDY [12] for solids and liquids under periodic boundary conditions; or GULP [13] for solids. Commercial suites include, among others, CHARMM [14], AMBER [15], and HyperChem [16].

With respect to Monte Carlo simulations, homemade software programs usually target a specific type of polymer structure, either of its chemistry or the architecture of the chain, but most of them follow rather similar approaches. Monte Carlo simulations are applied when equilibrium structural properties, including phase transitions, constitute the main research focus. The Enhanced Monte Carlo code [17,18] is a multi-purpose modular environment for particle simulations using force fields such as COMPASS, CHARMM, or Born. This open tool has been used to study the effect of semicrystalline interphase polyethylene under different conditions of tensile deformation [19,20] or chain branching [21]. MCCCS Towhee [22] was initially developed as a simulator suitable for computing phase equilibria in the Gibbs ensemble, but later extended to different force fields and ensembles. As an example, this open tool has been used to study gas-mixture separations on clathrate hydrates [23] among many other studies. DL_MONTE [24] is a very recent MC-based open tool that can be applied to general atomistic systems under different force fields and ensembles, as well as introducing transition pathways of umbrella sampling and Wang–Landau [25]. Furthermore, it is compatible with the molecular dynamic tool DL_POLY or chain branching [21].

We should also mention other relevant open-source MC-packages, such as Cassandra [26], that can be applied to obtain thermodynamic properties of fluids and solids; RASPA [27] for simulating adsorption and diffusion phenomena; GOMP [28,29] for GPU optimized phase equilibria simulations; or DICE [30] that uses a configurational bias scheme to study flexible molecules in solute-solvent systems. Most relevant MC software packages are benchmarked in terms of computational efficiency using adsorption simulations [31]. Regarding realistic polymeric systems, Chameleon [32] is one of the latest available pieces of software. This tool employs different chain connectivity altering moves to simulate atomistically detailed polyethylene (PE), polystyrene (PS), and polyvinyl chloride (PVC) for different polymer architectures.

Usually, the development of a commercial or open code, especially when built around Monte Carlo algorithms (moves), requires a major effort and programming in order to make it user-friendly, efficient, and of general applicability. Besides, it is very common that clever MC-based or general structure-optimization algorithms have and are being developed for specific applications or general classes of physical problems in continuous or lattice cells and in systems of varied chemical detail, in the bulk and under confinement [33,34,35,36,37,38,39,40,41,42,43,44,45,46,47,48,49,50,51,52].

Equally important to the simulation itself is the post-simulation analysis. This step can include visualization, including 3-D representation and animation, of the computer-generated system configurations and calculation of relevant quantities through proper interpretation of the raw simulation data. Corresponding suites also exist for interactive visualization, description, and analysis including, among others, ParaView [53], VMD [54], disLocate [55], UCSF chimera [56], OVITO [57], and i-Rheo GT [58].

In the present manuscript, we analyze the main features of Simu-D, an MC-based simulator and structural descriptor suite for the molecular modeling of polymer-based systems under extreme conditions. The simulator, which is the central component of the present software, is effectively the accumulation of successive expansions, modifications, and improvements implemented on the MC code [59], originally built for the simulation of dense and jammed athermal polymer-based systems in the bulk. The structural descriptor is the latest version of the Characteristic Crystallographic Element (CCE) norm [60,61], a metric used to gauge the similarity of local structure with respect to reference crystals in general atomic and particulate systems. Over the last years, the MC suite has been extended to simulate athermal polymers under confinement [62] and more recently macromolecules whose monomers interact with the square well (SW) or square shoulder (SS) potential [63]. In the corresponding research studies, emphasis was placed on how the employed conditions affect the ability of chains to pack at the local and global level [64,65], the topological network of entanglements [66,67,68], and the entropy- or energy-driven phase behavior (crystallization) in the bulk and under extreme confinement [63,69,70,71,72,73]. Here, the suite is further extended to include additional factors: chain stiffness, blends of chains and monomers, spherical or cylindrical confinement, the varied potential for bonded and non-bonded interactions, nanofillers in the form of cylinders and spheres, and combinations of the above. The ongoing effort is to create a general-purpose simulator-descriptor suite that will be as efficient, general, and user-friendly as possible given the variety of simulation conditions to be considered and the stochastic nature of the underlying MC method.

The manuscript is organized as follows: Section 2 presents the molecular model, the interspecies interactions, and the systems under study. Section 3 presents the moves behind the MC simulator and briefly discusses the features of the CCE-based structural descriptor. Section 4 discusses results from representative applications of Simu-D. Finally, Section 5 summarizes the main conclusions and lists current efforts and plans.

## 2. Molecular Model/Systems Studied

The current version of Simu-D allows the simulation of atomistic systems composed of *N*_at_ spherical monomers. These monomers can be part of macromolecules and/or exist as individual particles. In the general case, the system contains *N*_ch_ chains with the average length of *N* and *N*_s_ individual particles with *N*_ch_ × *N* + *N*_s_ = *N*_at_. Obviously, the two limiting cases correspond to the pure polymer matrix (*N*_s_ = 0, *N*_at_ = *N*_ch_ × *N*) and a system composed entirely of monomers (*N*_ch_ = 0, *N*_at_ = *N*_s_).

Non-bonded atoms interact with a pair-wise potential, which can be discontinuous such as the hard sphere (HS) or the square well/shoulder (SW/ SS) ones or continuous such as Lennard–Jones (LJ) with the corresponding formulas being displayed in Equation (1).

(1) $$U_{HS}\left(r_{ij}\right)=\left\{\begin{array}{c} 0,\;r_{ij}\geq \sigma \\ \infty ,\;r_{ij}<\sigma \end{array}\right.,\;U_{SW/SS}=\left\{\begin{array}{c} \;0,\;r_{ij}\geq \sigma_{2}\\ -\varepsilon_{SW},\;\sigma \leq r_{ij}<\sigma_{2}\\ \infty ,\;r_{ij}<\sigma \end{array}\right.,\;U_{LJ}=\varepsilon_{LJ}\left[{\left(\frac{\sigma_{LJ}}{r_{ij}}\right)}^{12}-{\left(\frac{\sigma_{LJ}}{r_{ij}}\right)}^{6}\right]$$

where *r_ij_* is the distance of the centers of atoms *i* and *j* and *σ* is the collision diameter, which is further considered as the characteristic length of the system. *σ*_2_ and *ε_SW_* correspond, respectively, to the range and intensity of the repulsive (SS) and attractive (SW) potentials. *ε_LJ_* and *σ_LJ_* are the depth and zero-energy point of the LJ potential. As in any traditional molecular simulation, depending on the type of the applied non-bonded potential, the original simulation cell is split automatically into overlap cells (HS), or into overlap and cut-off cells (SW/SS, LJ) to expedite the calculation of interactions.

Polymers are modeled as linear sequences of monomers of varying chain stiffness. Bond lengths can be longer (bond gaps), equal (bond tangency), or shorter (fused spheres) than the collision distance, *σ*. Chain stiffness is introduced through a potential governing bending angle (supplement of bending angle, *θ*) formed by triplets of successive atoms along the chain backbone. The formula for the energetic calculations can be general. Configurations of semi-flexible chains have been generated in the present work with the following bending angle potential:

(2) $$U_{bend}\left(\theta \right)=k_{\theta }{\left(\theta -\theta_{0}\right)}^{2}$$

where *k_θ_* is the bending constant and *θ*_0_ is the equilibrium bending angle supplement (i.e., a fully extended bending angle corresponds to *θ*_0_ = 0°). For fixed bond lengths, setting *k_θ_* = 0 allows the simulation of freely jointed chains while *k_θ_* → ∞ corresponds to the freely rotating model. In the current version of the suite, torsion angles, *φ*, can also be controlled through the implementation of a torsional potential, *U_tor_*(*φ*). However, in all results presented below, torsion angles are allowed to fluctuate freely and thus chain stiffness is governed solely by the bending potential.

The presence and activation of specific MC moves, as will be described in the continuation, enforces dispersity in chain lengths. Such polydispersity is controlled by casting the simulation in the N_at_N_ch_VTμ* ensemble where *V* is the total volume of the simulation cell, *T* is the temperature, and μ* is the spectrum of relative chemical potentials of all chain species, as explained in detail in References [59,74]. The uniform and Flory (most probable) distributions of molecular lengths can be selected in the simulation of polydisperse systems. In the case that strictly monodisperse samples are required, then all moves that vary the chain length (sEB, x-reptation, and IdEx3, see below) are deactivated from the mix.

Depending on the system under study, initial configurations are generated under very dilute conditions and the system is brought to the desired density through compressions or simulations in the isothermal-isobaric (*NPT*) ensemble. For the latter, conventional volume fluctuation moves are attempted at regular intervals. For the former, cell compaction is achieved by a combination of volume fluctuation moves, and in the case of confined systems, the wall wrapping “MRoB” algorithm as explained in [75].

Simulations can be conducted in two or three dimensions under periodic boundary conditions or on flat surfaces. Confinement is realized through the presence of such impenetrable surfaces. The current implementation allows confinement in the form of (i) flat, parallel walls in at least one dimension, (ii) a cylinder with closed or open ends (subjected to periodic boundary conditions), and (iii) a sphere (full confinement). The intensity of confinement is controlled by the distance between the confining surfaces, i.e., the cylinder or sphere diameter or the inter-wall distance. The latter can, in general, be different in each confined dimension *i*, *d_wall_*(*i*). Simulation cells are always orthogonal but can be anisotropic, and the number of confined dimensions, *d_conf_*, ranges from 0 (bulk cell with periodic boundary conditions) to 3 (full confinement). The cell aspect ratio, *ζ*, is defined as the ratio of the maximum inter-wall distance divided by the minimum one [75].

Nanocomposites can be simulated with the fillers taking the form of spherical or cylindrical particles of varied sizes and populations. In the current implementation of the suite, each nanocylinder spans the whole simulation cell and its direction is held fixed throughout the simulation. Nanospheres can, in principle, move in space, but in all computer-generated polymer nanocomposite configurations to be presented in the continuation, they are treated as immobile inclusions.

For a bulk system of pure polymer, the matrix number density, *ρ*, is trivially defined as $\rho =N_{at}/V$, while for non-overlapping entities (such as hard spheres), packing density, *ϕ*, is given by:

(3) $$\varphi =\frac{V_{mon}}{V}=\frac{\pi }{6}\frac{N_{at}}{V}\sigma^{3}=\frac{\pi }{6}\rho \sigma^{3}$$

where *V* is the volume of the simulation cell and *V_mon_* is the volume occupied by the monomers, either as individual entities (“single monomers”) or by being part of polymer chains (“chain monomers”).

For interfacial/confined/composite systems, the above definition provides little information on the free or accessible volume given that for very large nanofillers, the volume occupied by the nanofiller can be up to four orders of magnitude higher than the one of the monomers. Thus, we can further define an effective packing density, *ϕ_eff_*, considering the reduction of the accessible volume due to the presence of the nanofillers as:

(4) $$\varphi_{eff}=\frac{V_{mon}}{V_{acc}}=\frac{V_{mon}}{\left(V-V_{fill}\right)}$$

where *V*_acc_ is the volume accessible to the spherical monomers, *V_fill_* (= *V_cyl_* + *V_sph_*) is the volume occupied by the nanofillers, being the summation of the volume occupied by *N_cyl_* cylinders (*V_cyl_*) and of *N_sph_* spheres (*V_sph_*). Additionally, in the calculations above, one could further incorporate a depletion layer as monomer centers cannot lie closer than σ/2 from the surface of nanofillers or walls. In the general case of a system under confinement and being composed of nanofillers, if *d_conf_* is the number of confined dimensions, the depleted effective packing density, *ϕ_dep_*, including the effect of all nano-entities, can be calculated as:

(5) $$\varphi_{dep}=\frac{V_{mon}}{V_{dep}}=\frac{V_{mon}}{\left(\prod_{i=1}^{d_{conf}}\left(d_{wall}-\sigma \right)\prod_{j=d_{conf}+1}^{3}l_{j}-\frac{\pi }{6}{\left(d_{sph}+\sigma \right)}^{3}N_{sph}-\frac{\pi }{4}{\left(d_{cyl}+\sigma \right)}^{2}L_{cyl}N_{cyl}\right)}$$

where *d_sph_* and *d_cyl_* are the diameter of the nanospheres and nanocylinders, respectively, *L_cyl_* is the nanocylinder length, index *i* runs over all confined dimensions, index *j* over all unrestricted ones, and *l_j_* is the length of the simulation cell in dimension *j*.

## 3. Simulator-Descriptor Suite

### 3.1. Simulator

The Monte Carlo suite (“simulator”) consists of four different classes of algorithms: (1) Standard localized moves that entail the displacement of a single or a sequence of atoms, (2) chain-connectivity-altering moves (CCAMs), (3) cluster-based moves, and (4) identity exchange moves, all being executed at a constant volume. When shrinkage or NPT simulations are conducted, the regular volume fluctuation moves and/or the MRoB algorithm [75] undertake the task of changing the dimensions of the orthogonal simulation cell. This size alteration can be isotropic or anisotropic.

**Figure 1:**
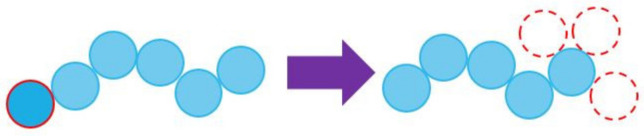
Schematic of the reptation move implemented through a configurational bias pattern with *n_trials_* = 3. Different candidate positions could be picked by the selection of the bond length, bending, and torsion angles used for the re-construction of the monomer.

The local moves have been described exhaustively in numerous past publications. For single monomers, the simplest possible move is that of a displacement in a random direction and length within a preset amplitude [0, *l_disp_*(*i*)], which again can be different for each dimension, *i*. With respect to chains, the corresponding set consists of: (i) Flip (internal libration), (ii) end-mer rotation, (iii) reptation, (iv) intermolecular reptation, and (v) end-segment re-arrangement (or CCB as in [76,77]; the reason we use a different notation here is to avoid confusion with the general scheme employed in all moves is explained next). All polymer-related moves can be executed in a configurational bias (CB) pattern (as seen in Figure 1 for the reptation move), with the number of trial configurations per attempted move, *n_trials_*, being an input variable in the simulator. Due to the introduction of energetic bias in the forward transition, the reverse transition must be attempted *n_trials_*-1 times to guarantee microscopic reversibility. In general, the number of attempts can be different for each local move, *n_trials_*(*i*), where index *i* runs over all available polymer-based moves. This is because the individual MC moves are characterized by distinctly different acceptance rates, which are further heavily affected by simulation conditions, chain stiffness and especially by concentration (packing density). As intuitively expected, increasing the number of trial configurations leads to a significant increase in the computational time required per MC move. Setting *n_trials_* = 1 enables the conventional execution of the local moves and eliminates the necessity to perform the reverse transition. The selection of *n_trials_* is highly system dependent; for example, optimal values for hard-sphere chains in the bulk as a function of the volume fraction from dilute conditions up to the maximally random jammed (MRJ) state can be found in Table 1 of Ref. [59].

The set of chain-connectivity-altering moves consists of the simplified end-bridging (sEB), simplified intramolecular end-bridging (sIEB), and simplified double bridging (sDB) [59,75] moves. All constitute simplified versions of the original EB [74,78] and DB [79,80] algorithms, initially developed for the rapid equilibration of atomistically detailed polyethylene chains of high molecular weight. The main difference with respect to the original moves is that none of the simplified versions entails the displacement of atoms; rather they proceed by deleting and forming properly selected bonds in a pair (sEB, sDB) of chains or a single (sIEB) chain. The main advantage of the sDB algorithm is that it can be applied to strictly monodisperse systems and primarily to non-linear molecular architectures. Its main disadvantage is that it requires a bridgeable distance between two different pairs of atoms. For systems of very small bond gaps (*dl* → 0), this condition is very rarely met except very near the jammed state where the contact network is rich as a result of the isostaticity condition [65]. Additionally, all systems to be reported in the continuation are composed of linear chains. Furthermore, it has been found that dispersity in chain lengths has no effect on the universal static scaling laws [66,67] and phase behavior [71,72] of the simulated thermal and athermal polymer packings. Based on the above, sDB is excluded from the mix of moves for all cases studied here.

**Figure 2:**
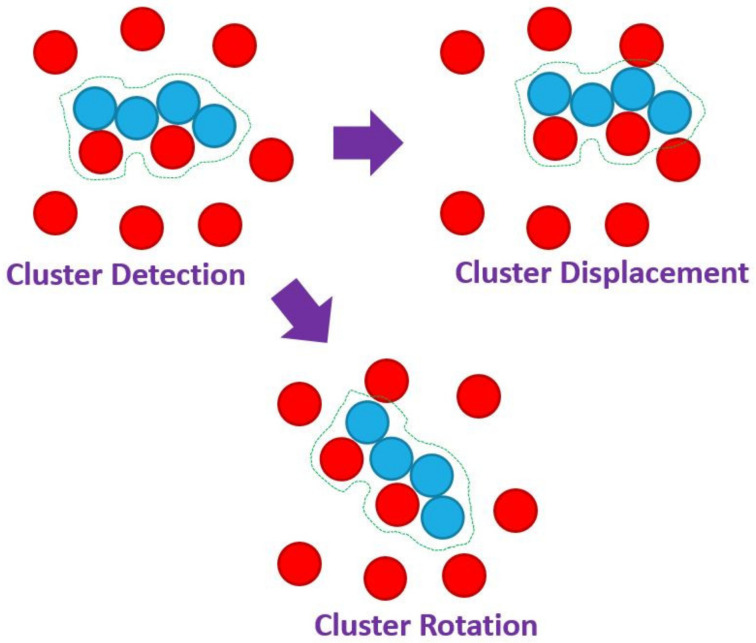
Schematic representation of the cluster displacement (CluDis) and cluster rotation (CluRot) moves in a mixed system of chain (**blue**) and single (**red**) monomers. The initial step of cluster detection is performed based solely on proximity criterion. The identified cluster is shown by the contour line. The cluster, as a whole unified group of monomers, can then be displaced in a random direction and length (ClusDis) or be rotated by a random amount around its center of mass (ClusRot).

The third class of MC moves is that of cluster-based ones. The two variations, implemented in Simu-D, are cluster rotation (CluRot) and cluster displacement (CluDis) as first introduced in the home-made cluster code reported in [63]. The execution of the moves proceeds according to the schematic in Figure 2. In the first step, the cluster is identified. Group similarity for cluster detection is conducted first through a Euclidean distance criterion, independently of the identity of the constituent atoms (chain versus single monomers etc.). Further linkage criteria can include additional common elements such as the same crystal similarity (as detected for example by the CCE analysis, see below). Once the clusters are identified with the corresponding members labelled accordingly, one cluster is selected randomly. That cluster, as a whole (i.e., a single object made of the corresponding sites), can be displaced by a random amount in a random direction (CluDis) or be rotated randomly with respect to its center of mass (CluRot). The cluster-related moves can be optionally and automatically de-activated when a single cluster exists in the system.

The cluster detection is a computationally demanding step, so the CluDis and CluRot moves have low attempt probabilities, as also happens with the chain-connectivity-altering ones and the algorithms that alter cell dimensions.

**Figure 3:**
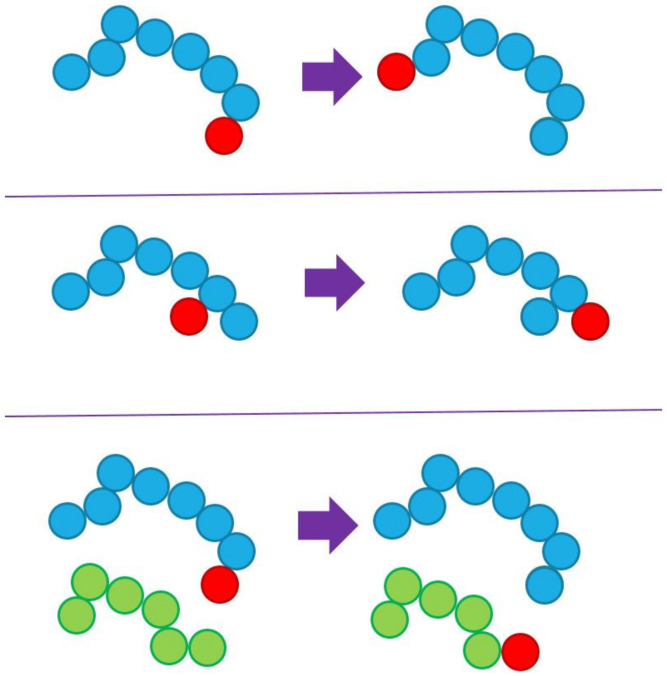
Schematic representation of (**top**) IdEx1 involving a chain and a single monomer within a bridgeable distance from a chain end, (**middle**) IdEx2 involving a chain and a single monomer within a bridgeable distance from the atom lying in the second of penultimate position in the chain, and (**bottom**) IdEx3, which includes a pair of chains and a single monomer. In IdEx3, the single monomer, lying within a bridgeable distance from an end of the blue chain becomes part of it, while a randomly selected chain (shown here in green) loses a randomly selected end, which becomes a single monomer. None of the moves depicted above include the displacement of atoms, rather only deletion and formation of bonds. IdEx3 requires dispersity in chain lengths in order to be applicable. In all cases, chain monomers are shown in blue (or green) and single monomers in red.

The fifth and final set of moves consists of algorithms that change the identity of atoms and can be applied in the case of blends of monomers and polymers but also of polymers composed of different monomers. Figure 3 presents three such identity exchange (IdEx) moves, involving a single monomer and a single chain or a pair of chains. In the top panel of Figure 3, the execution of IdEx1 is shown once a single monomer (shown in red) is within a bridgeable distance to one of the ends of the chain molecule (shown in blue). The move proceeds by connecting, via a bond, the chain end and the single monomer so that the newly incorporated atom becomes the new chain end. In parallel, the last bond connecting the other end of the chain is deleted and the end is converted to a single monomer. By construction, the move does not entail atom displacement but rather the reconstruction of properly selected bonds. Accordingly, the change in energy entering the Metropolis criterion for acceptance or rejection of the move is due to the bonded term (variation of one bond length, one bending, and one torsion angle), along with any non-bonded change due to the swap of identities. The concept of IdEx2 (middle panel) is very similar. The single monomer needs to be within a bridgeable distance from the second or penultimate atom of the chain. If the proximity condition is fulfilled, it becomes, through bond formation, the new chain end, and the corresponding chain end is converted into a single atom through bond deletion. Finally, IdEx3 (bottom panel) entails two chains. The difference with respect to the single-chain version is that the new single monomer is created by the deletion of a terminal bond of a randomly selected chain, different than the one that gains the monomer. Clearly, the implementation of IdEx3 requires dispersity in chain lengths.

Based on the concept of identity exchange, as presented above, one can envision variations with monomers being incorporated into the inner segments of the polymer chains. However, such an approach would require the double fulfillment of the bridgeable distance and would therefore significantly reduce the pair of sites that could trigger such IdEx moves. For this reason, no further modifications have been incorporated in the present implementation of the simulator.

### 3.2. Descriptor

As mentioned earlier, equally important to the simulation itself is the analysis of the results, which can be “on the fly” or in a post-simulation step. Monte Carlo simulations, such as the ones presented here, provide no dynamical information, so the emphasis is placed on the study of the local and global structure, organization, topology, and phase behavior. Over recent decades, conceptually different approaches have led to the development and application of descriptors and analyzers of local structure in computer-generated configurations or of digitally processed experimental samples [81,82,83,84,85,86,87,88,89,90,91,92,93].

Here, since we are particularly interested in studying entropy- and energy-driven crystallization of polymer-based systems under extreme conditions, we propose a modeling scheme where the MC-based simulator is connected to a descriptor of the local structure (“descriptor”) in the form of the CCE norm [60,61]. The version adopted in Simu-D is very similar to the one we presented very recently, so the concept, methodology, and technical implementation, reported in detail in [60], are all also applicable to the present context. Thus, in the continuation, we will provide a brief description on the main aspects of the CCE norm descriptor and the new features, as implemented in Simu-D. Given an atomic or particulate system in two or three dimensions, the CCE norm proceeds by comparing the local environment around each site with the ideal ones of specific reference crystals.

The main concept behind the CCE norm descriptor is that each ideal crystal is uniquely identified by a set of symmetry operations (elements of its point group) [94,95,96,97]. The identification of the totality of these crystallographic operations, or of an equally discriminating subset of them, and their application to the nearest neighbors of an atom or particle is key in the implementation of the CCE algorithm.

As explained in detail in Ref. [60], the CCE norm is defined with respect to a specific crystal *X*. Once the reference crystal *X* is selected, the point group is identified along with the generating symmetry elements. Given a site (atom or particle), *i*, the *N_vor_*(*i*) nearest neighbors are identified through Voronoi tessellation. The *N_vor_*(*i*) population is then compared against the coordination number of the reference crystal *X*, *N_coord_*(*X*). The latter is, for example, equal to 12 for the face-centered cubic (FCC) and hexagonal close-packed (HCP) crystals in 3-D and 6 for the triangular (TRI) crystal in 2-D. If the coordination number is larger than the number of nearest neighbors, *N_coord_*(*X*) > *N_vor_*(*i*), a penalty function is applied [60]. In the opposite case, *N_coord_*(*X*) < *N_vor_*(*i*), only the *N_coord_*(*X*) closest neighbors are kept for the successive CCE-based analysis. The characteristic crystallographic element(s) is(are) identified and the corresponding actions for each one of them are applied to the coordinates of the neighbor atoms relative to the given site. For example the HCP crystal, with *N_coord_*(HCP) = 12, has one geometric symmetry element in the form of a sixfold roto-inversion axis, while the body centered cubic (BCC), with *N_coord_*(BCC) = 8, has five such elements: Four three-fold roto-inversion axes and one inversion center.

One important point in the CCE norm analysis in a 3-D system is that the orientation of each symmetry axis, or at least of a sub-set of them, is not known a priory. Accordingly, we scan the orientation space SO(3) around the given site with a mesh of discretization width *ϕ_step_*, which is the same for the azimuthal and polar angles.

For a given orientation, the actions of the symmetry element are executed. This procedure is then repeated over all symmetry elements. The goal of these crystallographic operations is to map the real coordinate system (given site *i* and *N_coord_*(*X*) neighbors) into the ideal one of the reference crystal *X*. Once this is completed, the algorithm proceeds to the next point of the discrete mesh until the whole orientation space is examined. This mapping allows to simultaneously quantify the orientational and radial similarity of the given local environment with respect to the ideal one of crystal *X*. This is realized through the calculation of a norm (see Equation (2) of [60]). The CCE-based norm for the given atom *i* with respect to reference crystal *X*, $\varepsilon_{i}^{X}$, is the one that corresponds to the global minimum of the norms as calculated over all possible orientations of all symmetry elements (axes). The same process is repeated over all particles or atoms of the systems and all reference crystals. Currently, the CCE descriptor, as implemented in Simu-D, includes the following crystals: Face-centered cubic (FCC), hexagonal close-packed (HCP), body-centered cubic (BCC), and hexagonal (HEX) for 3-D systems and honeycomb (HON), square (SQU), and triangular (TRI) for 2-D systems. Additionally, the local structure can be quantified with respect to fivefold (FIV) and pentagonal (PEN) local symmetries, in 3-D and 2-D, respectively. The lower the value of the CCE norm, the higher the similarity of the local environment to the reference crystal. A site is labelled *X*-type when its minimum CCE norm is lower than a critical threshold, *ε^thres^*, i.e., $\varepsilon_{i}^{X}\leq \varepsilon^{thres}$. By construction, as the characteristic crystallographic elements and operations constitute a distinctive feature for a crystal, the CCE norm is highly discriminatory, so that when the CCE norm with respect to crystal *X* is low, the corresponding norm for other crystal types is high.

An extensive analysis of the underlying concept, the minimum distinguishable set of symmetry elements and corresponding actions for each reference crystal, the algorithmic implementation on the CCE-norm descriptor, the required computational time, and the optimal selection of parameters are all discussed in detail in Ref. [60]. The Simu-D version contains certain additional features. As an option, the “on-the-fly” implementation allows, during the scanning of the spherical space, for the CCE analysis to stop when the norm is found to be lower than the pre-set threshold and pass to the next atom so as to expedite the process and provide a preliminary structural identification. Additionally, the CCE descriptor further identifies the clusters of all atoms that bear the same similarity. For example, it detects clusters of ordered sites, calculates their size (in number of atoms), as well as their shape. The cluster-based analysis functions with the same proximity criterion as the cluster identification used for the moves of the simulator component. An additional condition for the cluster identification is that it should contain sites that have all the same similarity (with respect to a single crystal type *X* or to a pair of them (*X* or *Y*)). Finally, the CCE descriptor provides information on the shape, size, and statistics of the Voronoi cells, as extracted from the Voronoi tessellation.

A table with a summary of the main variables used by the Simu-D suite along with a brief description can be found in Appendix A (Table A1). 

## 4. Simu-D: Applications

In this section, we briefly present polymer-based systems that can be simulated and successively analyzed with the Simu-D suite. Emphasis is placed on the simulation of systems under extreme conditions: These can range from a very high concentration (packing density), extreme confinement, or presence of nanofillers with dimensions significantly larger than the monomer size or any combination of the above. In the case of entropy- or energy-driven phase transitions, the corresponding analysis takes place through the CCE descriptor on the frames and trajectories generated by the simulator component.

The main point to be highlighted is that the Simu-D suite is built in a modular-based approach with the goals of general applicability and simplicity. So, all examples in the continuation have been or can be simulated and successively analyzed without any modification of the code being required from the end user. Here, it is not our intention to exhaustively analyze the physical behavior of each reported case but rather to provide evidence that such systems can be modeled and then characterized by the Simu-D software.

### 4.1. Packing Efficiency of Semi-Flexible Athermal Polymers (3-D)

How atoms, particles, or macroscopic objects are packed in the most efficient way is a topic of paramount importance in various fields and applications. Ordered packings of non-overlapping spheres in 3-D have an upper limit in the volume fraction, which corresponds to the one reached by the HCP or FCC crystals [98,99]. For disordered systems of the same entities, the corresponding packing density is globally accepted to be approximately 10–12% lower and corresponds to the Random Close-Packed (RCP) limit [100,101] or its equivalent Maximally Random Jammed (MRJ) state [102]. In the very first application of the MC-based code that served as the initial seed for the Simu-D suite, it was demonstrated that freely jointed chains of tangent hard spheres can be packed as efficiently as monomeric analogs [103]. However, the corresponding state of semi-flexible polymers or even of freely rotating chains is still a subject for investigation [104,105]. To this end, we used the simulator component to generate and successively equilibrate random packings of semi-flexible chains with a varied equilibrium angle, degree of stiffness, as quantified by the spring constant in Equation (2), average chain length, and volume fraction. Exploring the combined effect of the physical variables stated previously requires the conduction of numerous simulations starting from dilute systems all the way up to the RCP/MRJ limit. The range of the latter is expected to be a function of the rigidity of the chain and thus depend strongly on the bending constant and equilibrium angle [104].

**Figure 4:**
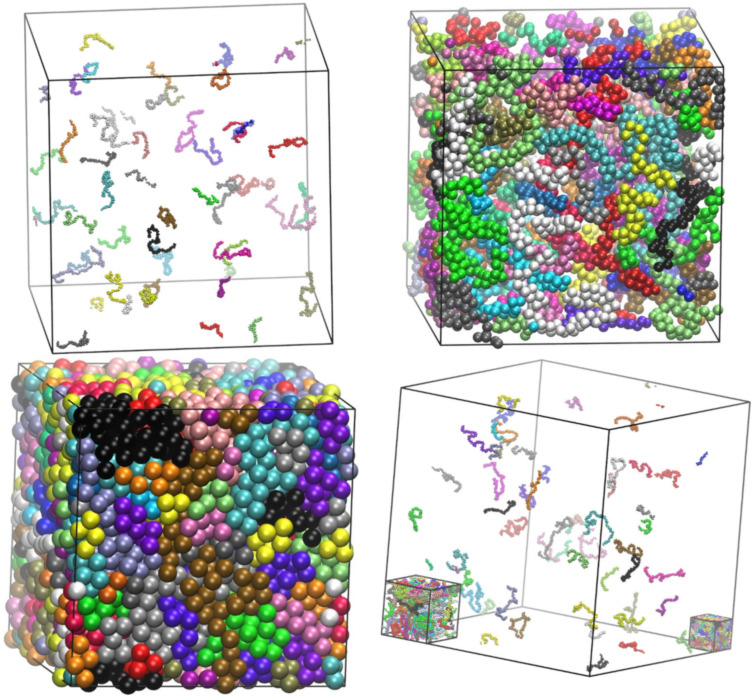
Bulk system configurations of semi-flexible chains of tangent hard spheres of uniform size with average length of *N* = 100 and an equilibrium angle of *θ*_0_ = 120° at progressively higher volume fractions, *ϕ*: (**top left**) 0.001, (**top right**) 0.10 and (**bottom left**) 0.60. (**bottom right**): All three system configurations shown together allowing for a visual comparison of their dimensions. Monomers are colored according to their parent chain. Sphere monomers are shown with coordinates of their centers subjected to periodic boundary conditions. Image created with VMD visualization software [54]. Figure panels are also available as interactive, 3-D images in Supplementary Materials.

Figure 4 shows bulk system configurations for semi-flexible hard-sphere chains with average length *N* = 100 (*N_at_* = 4800) with an equilibrium (supplement) angle of *θ*_0_ = 120° at different packing densities of *ϕ* = 0.001, 0.1, and 0.60.

**Figure 5:**
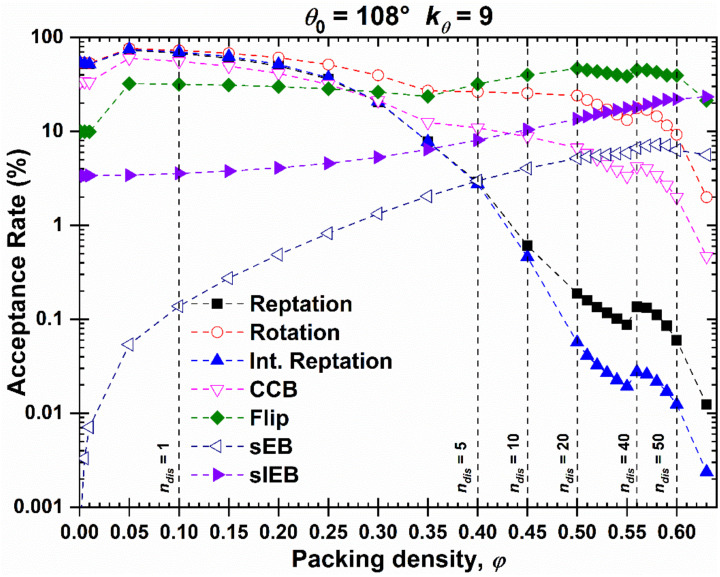
Percentage of acceptance as a function of packing density for the local and chain-connectivity-altering moves employed in the MC simulation of 48 chains of *N* = 100, *θ*_0_ = 108° in the bulk. Vertical dash lines denote the end of the regime where *n*_dis_ trial configurations are attempted per local MC move.

Using the Simu-D generation-equilibration modules, structures of semi-flexible athermal polymers can be simulated at very high densities, which are comparable to the densest ones observed for fully flexible (freely jointed) polymers [66,103] or monomeric counterparts [102]. The acceptance rate of the employed local and chain-connectivity-altering move as a function of packing density for the 48-chain *N* = 100 system with *θ*_0_ = 108° is shown in Figure 5 and is reminiscent of the one obtained for freely jointed chains [59]. As expected, the acceptance rate of local moves is significantly reduced as the system reaches progressively higher concentrations. Towards this, the configurational bias scheme aids in reducing this drop. The reduction for semi-flexible chains is especially apparent for the two variants of the reptation move. In sharp contrast, the acceptance rate of chain-connectivity-altering moves shows opposite trends: The higher the concentration, the higher the acceptance rate. Especially for the simplified End-Bridging at low volume fractions, acceptance is very small. This is expected as in such a dilute system there are very few or no pairs of atoms that can trigger the move. As the concentration increases, the population of such pairs also increases because chains start to feel each other and the contact network around each site becomes richer. In parallel, none of the CCAMs, as incorporated in Simu-D, requires the displacement of any atoms. Thus, their performance is enhanced at very high packing densities, and especially near the MRJ state.

According to the RCP/MRJ definition, the maximum-density state should correspond to the densest structures, which are characterized by the maximum randomness or, equivalently, the minimum order [102]. The concept of rattlers [102] and flippers [103] can be invoked to quantify the fraction of individual sites and groups of them, which are able to perform movements in their local vicinity for monomeric and polymeric packings, respectively. In both cases, it is well demonstrated that the flipper/rattler population diminishes as we approach the MRJ state. Alternatively, one could attempt to quantify the lack of order in the system through the proper definition of corresponding parameters. Towards this, we employ the CCE norm (descriptor module of Simu-D) to calculate the similarity of the local environment around each monomer site to the close-packed (HCP and FCC) crystals, which are the dominant ones in the crystallization of hard sphere packings at high volume fractions [72,106]. The absence of such crystals should correspond to a highly disordered but densely packed medium near or at the RCP/MRJ state.

**Figure 6:**
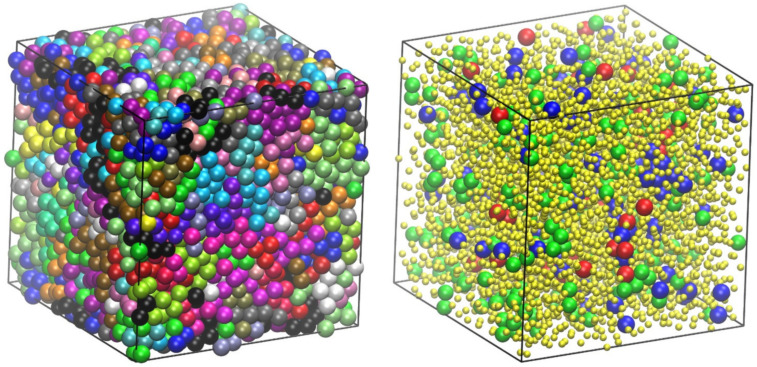
Jammed packing of semi-flexible chains of tangent hard spheres of uniform size with average length of *N* = 100 and an equilibrium angle of *θ*_0_ = 120°at a packing density of *ϕ* = 0.637. (**Left panel**): Monomers are colored according to the parent chain; (**Right panel**): Monomers are colored according to the lowest value of the CCE norm. Blue, red, and green denote HCP, FCC, and FIV similarity, respectively. Amorphous (AMO) ones are colored yellow with reduced dimensions for clarity. Sphere monomers are shown with coordinates of their centers being subjected to periodic boundary conditions. Image created with the VMD visualization software [54]. Figure panels are also available as interactive, 3-D images in Supplementary Materials.

Figure 6 shows the final configuration for the 48-chain *N* = 100 system with an equilibrium bending angle of *θ*_0_ = 120° at a density of approximately 0.64, which corresponds to the range of RCP/MRJ, as established for monomers and freely jointed chains. The left panel shows monomers colored according to the parent molecule, while the right one uses a coloring scheme according to the values of the CCE norm. More precisely, blue and red correspond to sites with HCP ($\varepsilon_{i}^{\mathrm{HCP}}\leq \varepsilon^{thres}=0.245$) and FCC ($\varepsilon_{i}^{\mathrm{FCC}}\leq \varepsilon^{thres}=0.245$) similarity, respectively, while green is used to represent FIV-like ($\varepsilon_{i}^{\mathrm{FIV}}\leq \varepsilon^{thres}=0.245$) sites. All remaining amorphous (AMO) ones, which constitute most of the system, are shown in yellow with reduced dimensions in a 2:5 scale for clarity purposes. More accurately, amorphous (AMO) designates sites that show no similarity to any of the reference 3-D crystal (HCP, FCC, HEX, BCC) or local symmetry (FIV). This does not exclude the possibility that a specific site showing similarity to another “unknown” crystal not included in the reference list. Still, as mentioned earlier and given the very high concentration of the generated athermal packings, the presence of non-compact crystals can be excluded. This is evident as no traces of BCC or HEX crystals are detected in any of the nearly jammed polymer configurations, such as the ones visualized in Figure 6.

Visual inspection of the jammed configuration in Figure 6 suggests a predominantly amorphous structure with few ordered HCP and FCC sites randomly distributed along the whole volume of the simulation cell. In fact, one can observe that the population of FIV-like sites is higher than that of the close packed crystal ones. Moving on to quantitative analysis based on the CCE order parameter [60] for the specific structure shown in Figure 6, the HCP, FCC, and FIV fractions are 0.022, 0.021, and 0.053, respectively, further demonstrating the predominance of disorder. The random character of the maximally jammed state for semi-flexible chains is in perfect qualitative agreement with the one exhibited by freely jointed analogs; the same can be stated for the growth of fivefold local symmetry with an increasing concentration as observed for monomeric counterparts [107,108] as well as for freely jointed chains [70].

### 4.2. Entropy-Driven Crystallization of Semi-Flexible Athermal Polymers

**Figure 7:**
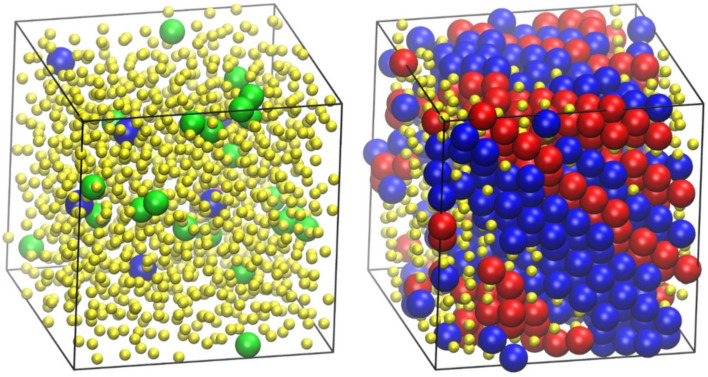
Snapshots of the semi-flexible *N* = 12 system (*θ*_0_ = 90°) at *ϕ* = 0.58. (**Left panel**): Initial configuration as produced by the generator module of Simu-D. (**Right panel**): Final configuration of the simulation after the execution of 3 × 10^11^ MC steps of the simulator module. Monomers are colored according to the lowest value of the CCE norm (descriptor module). Blue, red, and green colors denote HCP, FCC, and FIV similarity, respectively. Amorphous (AMO) ones are colored yellow with reduced dimensions for clarity. Spherical monomers are shown with coordinates of their centers subjected to periodic boundary conditions. Image created with the VMD visualization software [54]. Figure panels are also available as interactive, 3-D images in Supplementary Materials.

The presence of fivefolds in a random particulate packing acts as an inhibitor to crystallization [107,108], especially as the concentration approaches that of the jamming state. However, after a critical volume fraction (melting point) is exceeded, and if the observation (here simulation) time is sufficiently long, hard sphere packings crystallize. Similar phase behavior is observed for freely jointed chains, albeit with differences in the critical packing density and the morphology of the established crystals, both depending strongly on the gaps between bonded atoms [69]. Using the Simu-D suite we can extend the simulations to capture the effect of chain stiffness. As an example, Figure 7 shows the phase transition as first simulated and then identified by the CCE-based analysis for the 100-chain *N* = 12 system at *ϕ* = 0.58 with an equilibrium angle of *θ*_0_ = 90°. In the left panel of Figure 7, the initial configuration is presented, as produced through the generation module, while in the right panel, the final one after the execution of 3 × 10^11^ MC steps is shown. In both system states, monomers are colored according to the value of the CCE norm. It can be unmistakably concluded that the specific system shows crystallization, with the final stable configuration being of defect-ridden, fivefold-free, alternating HCP and FCC layers. Given that the hard-sphere chain system is athermal, such a phase transition is dictated solely by the increase in the total entropy of the system through a mechanism similar to the one observed in freely jointed chains where the local environment around each ordered site becomes more symmetric in the crystal phase [71,72,109].

### 4.3. Phase Behavior of Athermal Blends (Polymers and Monomers)

**Figure 8:**
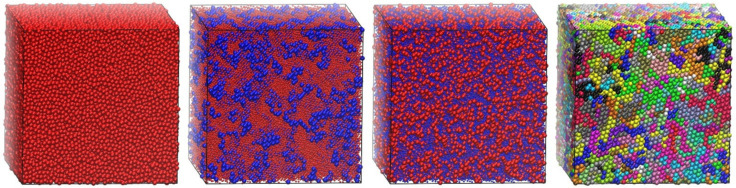
Bulk systems of 54,000 interacting hard spheres with varied relative concentrations of polymer content with chains having an average length of *N* = 1000 at *ϕ* = 0.58. The polymer relative concentration ranges from 0, 0.185, 0.741 to 1 (from left to right). In the pure polymer configuration (rightmost panel), sites are colored according to the parent chain. In all other cases, single and chain monomers are shown in red and blue, respectively. For chain concentrations of 0.185 and 0.741, sphere dimensions of dominant species are shown in a 2:5 scale for clarity. Image created with the VMD visualization software [54].

Through the incorporation of MC moves involving individual monomers and polymer chains (IdEx1, IdEx2, and IdEx3), Simu-D software can tackle blends of chains and monomers of varied relative fractions and different interactions between species. Example cases include a high-density athermal blend of polymers and monomers with a varied number of chains as can be seen in the panels of Figure 8. The system consists of 54,000 interacting sites at a packing density of *ϕ* = 0.56 and an average chain length of *N* = 1000. The polymer relative concentration ranges from 0 (0 chains and 54,000 monomers), 0.185 (10 chains and 44,000 monomers), 0.741 (40 chains and 14,000 monomers) to 1 (54 chains and 0 monomers). The objective here is to study how the relative concentration of the different entities (single versus chain monomers) could affect crystallization. This is motivated by the fact that the selected volume fraction is below and above the melting point of strictly tangent chains and individual monomers, respectively.

### 4.4. Energy-Driven Cluster and Crystal Formation of Attractive Chains

**Figure 9:**
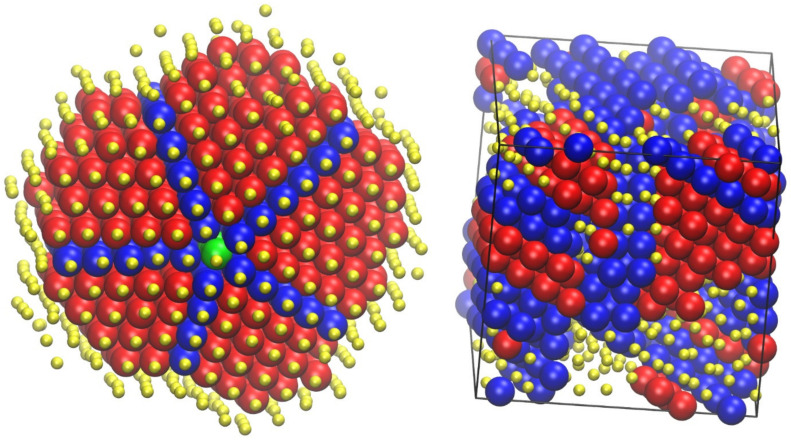
Final configurations of systems whose sites interact with the attractive square well potential. (**Left panel**) NVT simulations on chains (*ε*_SW_ = 1.2, *σ*_2_ = 1.15, *N* = 12, *N*_ch_ = 100, *ϕ* = 0.05); (**Right panel**) NPT simulations on monomers (*ε*_SW_ = 2.1, *σ*_2_ = 1.15, *N*_at_ = 1200). Sites are colored according to the CCE norm: Blue, red, green, cyan, and purple correspond to sites with HCP, FCC, FIV, BCC, and HEX similarity, respectively. Amorphous (AMO) sites are colored yellow and shown with reduced dimensions for clarity. Image created with the VMD visualization software [54]. Figure panels are also available as interactive, 3-D images in Supplementary Materials.

Up to this point, all systems studied were athermal with all interactions being of the hard sphere type. In the following case, we employ the square well (SW) attractive potential. Additionally, we carry out the simulations at a constant volume (NVT) for chain systems and at a constant (and high) pressure (NPT) for monomeric ones. In both cases, the starting configuration corresponds to a low-density (*ϕ* = 0.05) hard sphere system where we activate the SW interactions between all sites. Given the attraction felt between the monomers, clusters start to form, which, depending on the applied intensity and range of interactions, could further lead to ordered morphologies or amorphous glasses [63]. From the technical point of view in NVT simulations, one should activate collective, cluster-related moves since, especially at a low concentration and a high attraction intensity, small and isolated clusters could be created, disallowing further inter-cluster aggregation and the eventual formation of a single entity. The phase diagram of SW chains can be surprisingly rich with different crystals and amorphous morphologies resulting as a function of the attraction range. As an example, Figure 9 hosts the final system configuration obtained from NVT simulations on chains (*ε_SW_* = 1.2, *σ*_2_ = 1.15, *N* = 12) and NPT simulations on monomers (*ε_SW_* = 2.1, *σ*_2_ = 1.15), both having *N_at_* = 1200 interacting sites. For the given pairs of intensity and range of attraction, the established morphologies consist of HCP and FCC crystallites with random stacking directions. In the case of the chain cluster (left panel of Figure 9), the presence of fivefold sites in the form of twin defects is particularly evident in the meeting points of the HCP and FCC planes.

### 4.5. Polymers under Confinement

**Figure 10:**
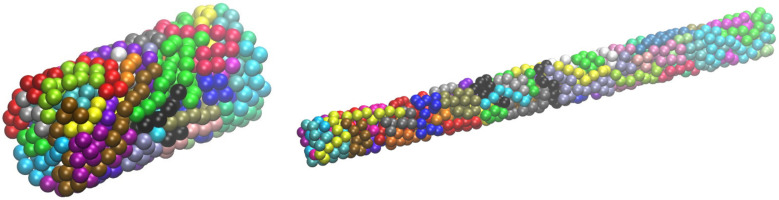
System snapshots of linear, fully flexible chains (*N*_ch_ = 60, *N* = 12, *ϕ* = 0.40) under cylindrical confinement with closed ends with a length-to-diameter ratio equal to 2 (**left panel**) and 10 (**right panel**). Monomers are colored according to the parent chain. Image created with the VMD visualization software [54]. Figure panels are also available as interactive, 3-D images in Supplementary Materials.

**Figure 11:**
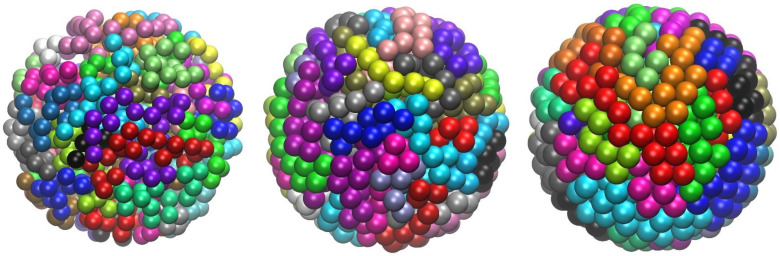
System snapshots of linear, fully flexible chains (*N*_ch_ = 60, *N* = 12) under spherical confinement at a packing density of (from left to right): *ϕ* = 0.30, 0.40 and 0.50. Monomers are colored according to the parent chain. Image created with the VMD visualization software [54]. Figure panels are also available as interactive, 3-D images in Supplementary Materials.

In a recent publication [75], we demonstrated the ability of the early version of the Simu-D suite to create polymer configurations under tube-like and plate-like confinement. Extreme conditions correspond to the case where the distance between the confining agents/surfaces is approximately equal to the diameter of the monomers. For example, for plates, this extreme condition corresponds effectively to a 2-D polymer system. The latest implementation of Simu-D allows for flexibility in the applied geometry of confinement departing potentially from orthogonal cells. Figure 10 and Figure 11 show system configurations of freely jointed chains of tangent hard spheres (*N* = 12, *N_ch_* = 60) being confined in cylindrical (closed ends) and spherical geometries, respectively.

In the cylindrical confinement, the cell (length to diameter) aspect ratio increases from 2 (left panel) to 10 (right panel) while the volume fraction remains the same (*ϕ* = 0.40). In the spherical one, the packing density changes from 0.30 (leftmost panel) up to 0.50 (rightmost panel). Reaching such densities allows the investigation of crystal nucleation and the growth of chain systems and eventually the comparison with monomeric analogs, as recently reported in [110,111].

### 4.6. Polymer Nanocomposites

**Figure 12:**
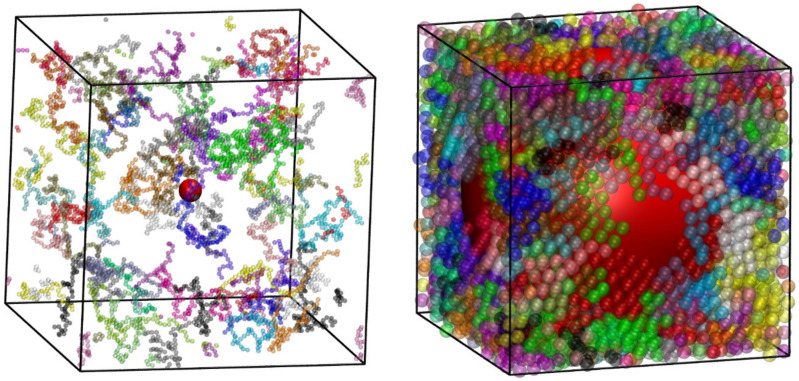
System snapshots of polymer nanocomposite (*N* = 100, *N*_ch_ = 48) at different effective packing density, *ϕ*_eff_. The nanofiller, shown in red, corresponds to a single, impenetrable sphere with diameter *d*_sph_ (in units of *σ*) whose center is located at the center of the simulation cell: (**left**) *ϕ*_eff_ = 0.01, *d*_sph_ = 5 and (**right**) *ϕ*_eff_ = 0.55, *d*_sph_ = 20. Monomers are colored according to the parent chain and are shown as semitransparent spheres for clarity. Image created with the VMD visualization software [54]. Figure panels are also available as interactive, 3-D images in Supplementary Materials.

The latest implementation of Simu-D allows for the simulation of polymer-based nanocomposites (PNCs). The nanofillers can exist as compact objects of cylindrical or spherical forms and at various concentrations and interactions with the chain monomers. Figure 12 shows examples of polymer nanocomposites where all entities interact through the hard-core potential. A single nanofiller is inserted, which is a sphere of size *d_sph_* (in units of monomer diameter, *σ*). The nanoparticle is positioned so that the coordinates of its center coincide with the center of the simulation cell. The left panel shows a PNC with *d_sph_* = 5 at *ϕ* = 9.9 × 10^−3^ while *d_sph_* = 20 at *ϕ* = 0.29 is presented in the right panel. Taking into account the presence of the nanosphere, the effective packing densities are *ϕ_eff_* = 0.01 and 0.55 for the systems in the left and right panels of Figure 12, respectively. The minimal difference for the former case is due to the small nanoparticle size (*d_sph_* = 5) compared to the large volume of the simulation cell, while in the latter case, the nanoparticle, due to its massive size (*d_sph_* = 20), has a profound effect on the reduction of the available volume.

**Figure 13:**
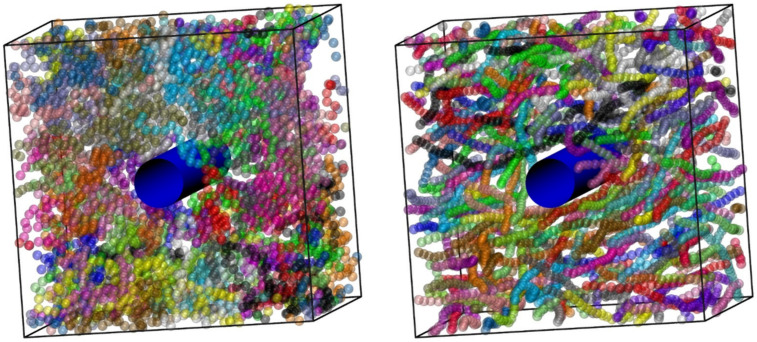
System snapshots of polymer nanocomposite (*N* = 100, *N*_ch_ = 48, *ϕ*_eff_ = 0.10). The nanofiller, shown in blue, corresponds to a single, impenetrable cylinder with diameter *d*_cyl_ = 5 (in units of σ) and infinite length. The cylinder is oriented along the direction of one of the cell axes. (**left**) Freely jointed chains and (**right**) semi-flexible, rod-like chains (*θ*_0_ = 0°). Monomers are colored according to the parent chain and are shown as semitransparent spheres for clarity. Image created with the VMD visualization software [54]. Figure panels are also available as interactive, 3-D images in Supplementary Materials.

Another example from MC simulations on PNCs is provided in Figure 13. This time the nanofiller takes the form of a single, infinitely long cylinder whose direction coincides with the direction of one of the axes of the simulation cell. The diameter of the cylinder is *d_cyl_* = 5 and is dispersed in a polymer matrix (*N* = 100, *N_ch_* = 48), which consists of (left panel) freely jointed and (right panel) semi-flexible, rod-like (*θ*_0_ = 0°) chains.

### 4.7. Comparison with Independent Algorithms

A relevant task is to compare the results of any simulation suite with existing ones, preferably using different simulation methods. Here we should mention that with respect to jamming, our Simu-D produces very dense and nearly jammed random packings of hard spheres (chains or monomers) with volume fractions very close to the ones reported in the literature from independent sources on the RCP/MRJ state: *ϕ*^MRJ^ ≈ 0.64–0.65, with the exact value and the salient characteristics being very dependent on the employed protocol [112,113,114].

In parallel, the melting point of monomeric hard spheres, as determined by simulations conducted with the present protocol, coincides with the well-established value available in the literature [115]. With respect to the crystallization of athermal polymers and the effect of bond gaps (or bond tangency), the results based on the application of the Simu-D suite [69] are in excellent agreement with independent studies using event-driven Molecular Dynamics (MD) simulations [116].

**Figure 14:**
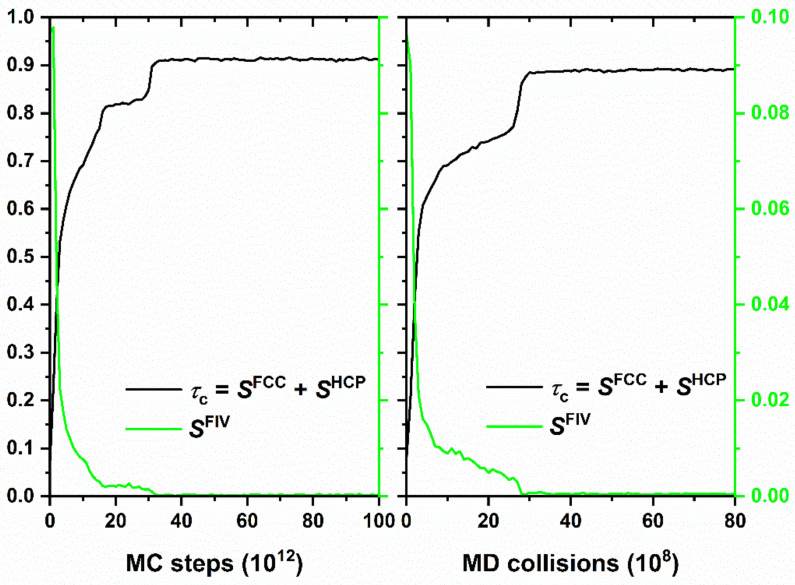
Evolution of crystallinity, *τ*_c_, and of the fraction of sites with fivefold (FIV) local symmetry, *S*^FIV^, as a function of MC steps (**left panel**) and MD collisions (**right panel**). Both the MC simulation, performed through the Simu-D suite, and the independent, event-driven MD simulation, are conducted on the same random initial configuration of 54,000 monomeric hard spheres of uniform size at a packing density of *ϕ* = 0.56. Total crystallinity is calculated here as the sum of fractions of sites with FCC and HCP character, as quantified by the CCE norm descriptor.

Furthermore, as an additional testbed, we use the crystallization of monomeric hard spheres. Towards this, we use the same amorphous system configuration, composed of 54,000 hard spheres at a volume fraction of *ϕ* = 0.56. Using this initial structure, we embark on two different kinds of simulation: (i) Stochastic MC using the Simu-D suite and (ii) event-driven, collision-based MD. Given that the reference system consists of hard spheres at an elevated concentration, total crystallinity can be considered as the sum of the fractions of sites with HCP and FCC-like similarity, with FIV local symmetry acting as a structural competitor to compact crystals. In both cases, the local structure is quantified through the CCE metric. Even if the two simulation methods are distinctly different, one (MD) based on collision-based dynamics the other (MC) being completely stochastic, the corresponding trends on the evolution of crystallinity, as seen in Figure 14, are strikingly similar, not only in qualitative but also in quantitative terms.

## 5. Conclusions

We present the latest implementation of Simu-D, a simulator-descriptor suite used to model and successively analyze/describe polymer-based systems under extreme conditions of concentration (packing density), confinement, and nanofiller content. The simulator part is based on Monte Carlo (MC) algorithms, including localized, chain-connectivity-altering, identity-exchange, and cluster moves in various statistical ensembles. The descriptor is based on the characteristic crystallographic element (CCE) norm, which is a metric to gauge the local structure by comparing it with reference crystals in two and three dimensions. The suite has a modular approach, allowing the addition of features, and is built considering efficiency, general applicability, and ease of use. Monomers/atoms/particles are presented as spheres, which interact through standard bonded and pairwise, non-bonded terms.

We have provided examples ranging from applications on bulk, pure macromolecular systems, of blends with monomers, under various conditions of confinement to polymer-based nanocomposites. Through such simulations one can study general phase transitions, packing ability, and local and global structure as a function of the aforementioned parameters. In the examples provided, emphasis is placed on the simplified hard-sphere and square-well models, but chemically realistic systems can be simulated as well.

**Figure 15:**
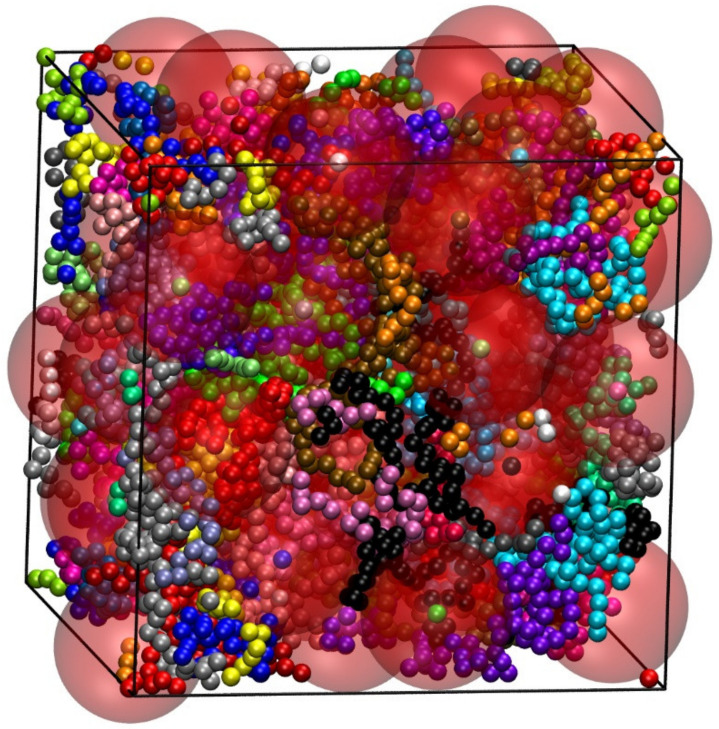
Terminally grafted nanoparticles on polymer chains at a volume fraction of *ϕ* = 0.50 as simulated through the Simu-D suite. Each nanoparticle, shown in red and in semitransparent format, has a size of *d*_nano_ = 8 and is anchored to a single polymer chain. Macromolecules are represented as freely jointed chains of tangent hard spheres with an average length of *N* = 100.

Presently, Simu-D is further expanded to tackle more complex systems including the simulation of terminally grafted nanoparticles anchored on polymer chains as seen in Figure 15, and polymer adsorption on flat or nanostructured surfaces.

Our simulator-descriptor suite is rather lacking with respect to the available potentials and interactions, especially compared to latest simulators [24]. However, as explained earlier, our intention is to simulate general, but still coarse-grained, representations of atomic and particulate systems with emphasis placed on extreme conditions, such as jamming, confinement, anchoring, presence of nanoparticles or all possible combinations of the above.

Chemical reactions can also be studied by assigning reactant and product types and implementing identity-change algorithms with the MC simulations being cast in the proper reactive ensemble [117].

In a coarse-to-fine approach, the present MC suite could be further benefited by algorithms that allow the simulation of chemically complex, all-atom systems through reversible, adaptive, or bijective mapping [34,35,36,37,118]. Furthermore, the suite should be compatible with independent and efficient MC algorithms such as the event-chain ones [40,41], parallel techniques [119,120], but also with different analyzers. The latter can be in the form of geometric [121,122], stochastic [123], or energy-based [124,125] codes for the topological analysis of the primitive path network of entanglements as abundantly encountered in densely packed systems of long polymer chains.

## Data Availability

Presented simulation trajectories and corresponding data are fully available upon request.

## Supplementary Materials

The following are available online at https://www.mdpi.com/article/10.3390/ijms222212464/s1. The manuscript is available in interactive, 3-D format. With the exception of Figure 8, all panels corresponding to system configurations are also available as stand-alone, interactive 3-D pdf files.

## Appendix A

The following Table is a summary of the main variables as used in the Simu-D suite for the simulation of different atomic systems (simulator part) and of the corresponding analysis of the local structure (descriptor part).

**Table A1:** Summary of the main variables as used by the Simu-D suite. Dashed line separates variables of the simulator and descriptor parts.

Name	Type	Description
*D*	Categorical	Number of dimensions
*dconf*	Categorical	Number of confined dimensions
*N_ch_*	Categorical	Number of chains
*N_at_*	Numerical	Total number of atoms
*N_high_*	Numerical	Maximum number of monomers per chain
*N_low_*	Numerical	Minimum number of monomers per chain
*N*	Categorical	Average number of monomers per chain
*N_mon_*	Numerical	Number of single monomers
*N_trials_*	Numerical	Number of trials per move in configurational bias scheme
*Opt_trials_*	Flag	Flag to select the density-dependence of *N_trials_*
*ccb_cut_*	Numerical	Maximum number of monomers moved in a CCB move
*disp*	Numerical	Maximum displacement of monomer moves
*φ*	Numerical	Packing density
*dl*	Numerical	Bond gap for chains
*Nanocomp*	Flag	Inclusion of nanoparticles
*N_cyl_*	Numerical	Number of nanocylinders
*N_sph_*	Numerical	Number of nanospheres
*d_cyl_*	Numerical	Diameter of nanocylinders
*d_sph_*	Numerical	Diameter of nanospheres
*dir_cyl_*	Array	Direction of nanocylinders
σ	Numerical	Diameter designation
$\sigma_{1}$	Numerical	Collision diameter for Square-Well/shoulder model
$\sigma_{2}$	Numerical	Range of interaction for Square-Well/shoulder model
ε	Numerical	Intensity of interaction for Square-Well/shoulder model
*Opt_SW_*	Flag	Creation of a second cell grid to improve SW performance.
$\varepsilon_{wall}$	Numerical	Intensity of interaction for Square-Well/shoulder of Walls
$\sigma_{2,wall}$	Numerical	Range of interaction for Square-Well/shoulder model of Walls
$\varepsilon_{part}$	Numerical	Intensity of interaction for Square-Well/shoulder of Nanoparticles
$\sigma_{2,part}$	Numerical	Range of interaction for Square-Well/shoulder model of Nanoparticles
*θ_eq_*	Numerical	Supplement of the equilibrium bending angle in radians
*k_bend_*	Numerical	Energy constant for bending angle potential
*NPT*	Flag	True: Enables NPT ensemble. False: Enables NVT ensemble
*T*	Numerical	Temperature
*P*	Numerical	Pressure
*Shrink*	Flag	True: Runs shrinkage production until a target density. False: Runs normal simulation
*fluc_vol_*	Numerical	Maximum box length reduction when attempting shrinkage
$\varphi_{target}$	Numerical	Target density for the shrinkage production
*Isotropic*	Flag	True: Volume changes are equal in all direction. False: Volume change is anisotropic
*Cluster*	Flag	Flag to enable cluster moves when there are more than one
*r_clust_*	Numerical	Radius to detect clusters
*Vec*	Flag	Storage of vectors for crystallographic elements
*Kiss*	Numerical	Coordination number of reference crystal
*Geom*	Flag	Check polymer geometry
*Neighs*	Numerical	Maximum number of Voronoi neighbors
*HCP*	Flag	CCE analysis for HCP crystal
*FCC*	Flag	CCE analysis for FCC crystal
*BCC*	Flag	CCE analysis for BCC crystal
*HEX*	Flag	CCE analysis for HEX crystal
*FIV*	Flag	CCE analysis for FIV symmetry
*HON*	Flag	CCE analysis for HON crystal
*SQU*	Flag	CCE analysis for SQU crystal
*TRI*	Flag	CCE analysis for TRI crystal
*PEN*	Flag	CCE analysis for PEN symmetry
*Thres*	Numerical	CCE threshold of similarity
*Step*	Numerical	Step of the mesh discretization (azimuthal and polar angles)
*Fast*	Flag	No full optimization if norm less than threshold
