# Supplementary material for: Simu-D: A Simulator-Descriptor Suite for Polymer-Based Systems under Extreme Conditions
Source: Int J Mol Sci. 2021 Nov 18;22(22):12464. doi: 10.3390/ijms222212464 (PMC8621175; doi:10.3390/ijms222212464)
Supplement: Supplementary file 1 [file ijms-22-12464-s001.zip › fig10a.pdf]

This area requires a 3D PDF enabled viewer such as Adobe Reader.

Figure 10a. System snapshots of linear, fully flexible chains (Nch = 60, N = 12, ◖ = 0.40) under cylindrical confinement with closed ends with a length-to-diameter ratio equal to 2. Monomers are colored according to the parent chain.
