# Supplementary material for: Simu-D: A Simulator-Descriptor Suite for Polymer-Based Systems under Extreme Conditions
Source: Int J Mol Sci. 2021 Nov 18;22(22):12464. doi: 10.3390/ijms222212464 (PMC8621175; doi:10.3390/ijms222212464)
Supplement: Supplementary file 1 [file ijms-22-12464-s001.zip › fig12b.pdf]

This area requires a 3D PDF enabled viewer such as Adobe Reader.

Figure 12b. System snapshots of polymer nanocomposite (N = 100, Nch = 48) at different effective packing density, ◖eff. The nanofiller, shown in red, corresponds to a single, impenetrable sphere with diameter dsph (in units of ů) whose center is located at the center of the simulation cell: ◖eff = 0.55, dsph = 20. Monomers are colored according to the parent chain and are shown as semitransparent spheres for clarity.
