# Supplementary material for: Simu-D: A Simulator-Descriptor Suite for Polymer-Based Systems under Extreme Conditions
Source: Int J Mol Sci. 2021 Nov 18;22(22):12464. doi: 10.3390/ijms222212464 (PMC8621175; doi:10.3390/ijms222212464)
Supplement: Supplementary file 1 [file ijms-22-12464-s001.zip › fig13a.pdf]

This area requires a 3D PDF enabled viewer such as Adobe Reader.

Figure 13a. System snapshots of polymer nanocomposite (N = 100, Nch = 48, ◖eff = 0.10). The nanofiller, shown in blue, corresponds to a single, impenetrable cylinder with diameter dcyl = 5 (in units of ů) and infinite length. The cylinder is oriented along the direction of one of the cell axes. Freely jointed chains. Monomers are colored according to the parent chain and are shown as semitransparent spheres for clarity.
