# Supplementary material for: Simu-D: A Simulator-Descriptor Suite for Polymer-Based Systems under Extreme Conditions
Source: Int J Mol Sci. 2021 Nov 18;22(22):12464. doi: 10.3390/ijms222212464 (PMC8621175; doi:10.3390/ijms222212464)
Supplement: Supplementary file 1 [file ijms-22-12464-s001.zip › fig13b.pdf]

This area requires a 3D PDF enabled viewer such as Adobe Reader.

Figure 13b. System snapshots of polymer nanocomposite (N = 100, Nch = 48, ◖eff = 0.10). The nanofiller, shown in blue, corresponds to a single, impenetrable cylinder with diameter dcyl = 5 (in units of ů) and infinite length. The cylinder is oriented along the direction of one of the cell axes.). Semi-flexible, rod-like chains (d0 = 0°). Monomers are colored according to the parent chain and are shown as semitransparent spheres for clarity.
