# Supplementary material for: Simu-D: A Simulator-Descriptor Suite for Polymer-Based Systems under Extreme Conditions
Source: Int J Mol Sci. 2021 Nov 18;22(22):12464. doi: 10.3390/ijms222212464 (PMC8621175; doi:10.3390/ijms222212464)
Supplement: Supplementary file 1 [file ijms-22-12464-s001.zip › fig15.pdf]

This area requires a 3D PDF enabled viewer such as Adobe Reader.

Figure 15. Terminally grafted nanoparticles on polymer chains at a volume fraction of ◖ = 0.50 as simulated through the Simu-D suite. Each nanoparticle, shown in red and in semitransparent format, has a size of dnano = 8 and is anchored to a single polymer chain. Macromolecules are represented as freely jointed chains of tangent hard spheres with an average length of N = 100.
