# Supplementary material for: Simu-D: A Simulator-Descriptor Suite for Polymer-Based Systems under Extreme Conditions
Source: Int J Mol Sci. 2021 Nov 18;22(22):12464. doi: 10.3390/ijms222212464 (PMC8621175; doi:10.3390/ijms222212464)
Supplement: Supplementary file 1 [file ijms-22-12464-s001.zip › fig4a.pdf]

This area requires a 3D PDF enabled viewer such as Adobe Reader.

Figure 4a. Bulk system configurations of semi-flexible chains of tangent hard spheres of uniform size with average length of N = 100 and an equilibrium angle of ɗ0 = 120° at ◖ = 0.001. Monomers are colored according to their parent chain. Sphere monomers are shown with coordinates of their centers subjected to periodic boundary conditions.
