# Supplementary material for: Simu-D: A Simulator-Descriptor Suite for Polymer-Based Systems under Extreme Conditions
Source: Int J Mol Sci. 2021 Nov 18;22(22):12464. doi: 10.3390/ijms222212464 (PMC8621175; doi:10.3390/ijms222212464)
Supplement: Supplementary file 1 [file ijms-22-12464-s001.zip › fig6a.pdf]

This area requires a 3D PDF enabled viewer such as Adobe Reader.

Figure 6a. Jammed packing of semi-flexible chains of tangent hard spheres of uniform size with average length of N = 100 and an equilibrium angle of đ0 = 120°at a packing density of ◖ = 0.637. Monomers are colored according to the parent chain. Sphere monomers are shown with coordinates of their centers being subjected to periodic boundary conditions.
