# Supplementary material for: Simu-D: A Simulator-Descriptor Suite for Polymer-Based Systems under Extreme Conditions
Source: Int J Mol Sci. 2021 Nov 18;22(22):12464. doi: 10.3390/ijms222212464 (PMC8621175; doi:10.3390/ijms222212464)
Supplement: Supplementary file 1 [file ijms-22-12464-s001.zip › fig6b.pdf]

This area requires a 3D PDF enabled viewer such as Adobe Reader.

Figure 6b. Jammed packing of semi-flexible chains of tangent hard spheres of uniform size with average length of N = 100 and an equilibrium angle of ɗ0 = 120°at a packing density of ◖ = 0.637. Monomers are colored according to the lowest value of the CCE norm. Blue, red, and green denote HCP, FCC, and FIV similarity, respectively. Sphere monomers are shown with coordinates of their centers being subjected to periodic boundary conditions.
