# Supplementary material for: Simu-D: A Simulator-Descriptor Suite for Polymer-Based Systems under Extreme Conditions
Source: Int J Mol Sci. 2021 Nov 18;22(22):12464. doi: 10.3390/ijms222212464 (PMC8621175; doi:10.3390/ijms222212464)
Supplement: Supplementary file 1 [file ijms-22-12464-s001.zip › fig7a.pdf]

This area requires a 3D PDF enabled viewer such as Adobe Reader.

Figure 7a. Snapshots of the semi-flexible N = 12 system (d0 = 90°) at ◖ = 0.58. Initial configuration as produced by the generator module of Simu-D. Monomers are colored according to the lowest value of the CCE norm (descriptor module). Blue, red, and green colors denote HCP, FCC, and FIV similarity, respectively.
