# Supplementary material for: Simu-D: A Simulator-Descriptor Suite for Polymer-Based Systems under Extreme Conditions
Source: Int J Mol Sci. 2021 Nov 18;22(22):12464. doi: 10.3390/ijms222212464 (PMC8621175; doi:10.3390/ijms222212464)
Supplement: Supplementary file 1 [file ijms-22-12464-s001.zip › fig7b.pdf]

This area requires a 3D PDF enabled viewer such as Adobe Reader.

Figure 7b. Snapshots of the semi-flexible N = 12 system (d0 = 90°) at ◖ = 0.58. Final configuration of the simulation after the execution of 3 × 1011 MC steps of the simulator module. Monomers are colored according to the lowest value of the CCE norm (descriptor module). Blue, red, and green colors denote HCP, FCC, and FIV similarity, respectively.
