# Supplementary material for: Simu-D: A Simulator-Descriptor Suite for Polymer-Based Systems under Extreme Conditions
Source: Int J Mol Sci. 2021 Nov 18;22(22):12464. doi: 10.3390/ijms222212464 (PMC8621175; doi:10.3390/ijms222212464)
Supplement: Supplementary file 1 [file ijms-22-12464-s001.zip › fig9a.pdf]

This area requires a 3D PDF enabled viewer such as Adobe Reader.

Figure 9a. Final configurations of systems whose sites interact with the attractive square well potential. NVT simulations on chains (ŮSW = 1.2, ů2 = 1.15, N = 12, Nch = 100, ◖ = 0.05). Sites are colored according to the CCE norm: Blue, red, green, cyan, and purple correspond to sites with HCP, FCC, FIV, BCC, and HEX similarity, respectively.
